# Short‐Term Ambient Air Pollution Exposure and Risk of Out‐of‐Hospital Cardiac Arrest in Sweden: A Nationwide Case‐Crossover Study

**DOI:** 10.1161/JAHA.123.030456

**Published:** 2023-10-11

**Authors:** Marcus Dahlquist, Viveka Frykman, Jacob Hollenberg, Martin Jonsson, Massimo Stafoggia, Gregory A. Wellenius, Petter L. S. Ljungman

**Affiliations:** ^1^ Institute of Environmental Medicine Karolinska Institutet Stockholm Sweden; ^2^ Department of Cardiology Danderyd University Hospital Sweden; ^3^ Department of Clinical Sciences Danderyd University Hospital, Karolinska Institutet Danderyd Sweden; ^4^ Center for Resuscitation Science, Department of Clinical Science and Education, Södersjukhuset Karolinska Institutet Stockholm Sweden; ^5^ Department of Epidemiology Lazio Region Health Service Roma 1 Italy; ^6^ Department of Environmental Health Boston University School of Public Health MA USA

**Keywords:** air pollution, case‐crossover design, out‐of‐hospital cardiac arrest, particulate matter, Sudden Cardiac Death, Cardiopulmonary Arrest, Risk Factors, Epidemiology

## Abstract

**Background:**

Air pollution is one of the main risk factors for cardiovascular disease globally, but its association with out‐of‐hospital cardiac arrest at low air pollution levels is unclear. This nationwide study in Sweden aims to investigate if air pollution is associated with a higher risk of out‐of‐hospital cardiac arrest in an area with relatively low air pollution levels.

**Methods and Results:**

This study was a nationwide time‐stratified case‐crossover study investigating the association between short‐term air pollution exposures and out‐of‐hospital cardiac arrest using data from the SRCR (Swedish Registry for Cardiopulmonary Resuscitation) between 2009 and 2019. Daily air pollution levels were estimated in 1×1‐km grids for all of Sweden using a satellite‐based machine learning model. The association between daily air pollutant levels and out‐of‐hospital cardiac arrest was quantified using conditional logistic regression adjusted for daily air temperature. Particulate matter <2.5 μm exposure was associated with a higher risk of out‐of‐hospital cardiac arrest among a total of 29 604 cases. In a multipollutant model, the association was most pronounced for intermediate daily lags, with an increased relative risk of 6.2% (95% CI, 1.0–11.8) per 10 μg/m^3^ increase of particulate matter <2.5 μm 4 days before the event. A similar pattern of association was observed for particulate matter <10 μm. No clear association was observed for O_3_ and NO_2_.

**Conclusions:**

Short‐term exposure to air pollution was associated with higher risk of out‐of‐hospital cardiac arrest. The findings add to the evidence of an adverse effect of particulate matter on out‐of‐hospital cardiac arrest, even at very low levels below current regulatory standards.

**Nonstandard Abbreviations and Acronyms**
OHCA: out‐of‐hospital cardiac arrest
PEA: pulseless electrical activity
PM_2.5_
: particulate matter with a diameter<2.5 μm, fine particles
PM_10_
: particulate matter with a diameter<10 μm
SRCR: Swedish Registry of Cardiopulmonary Resuscitation
WHO: World Health Organization

**Clinical Perspective**

**What Is New?**

- In this nationwide case‐crossover study in Sweden, a country with air pollution levels well below current European regulatory limits, exposure to particulate matter increased the risk of suffering a cardiac arrest.

**What Are the Clinical Implications?**

- The study provides additional evidence in support of the negative impact of air pollution exposure on health, both in urban and rural settings, and already at low concentrations of air pollutants.
- Future studies should investigate potential susceptible groups and the potential interactions between air pollution, temperature, and climate.

Air pollution is a leading risk factor for the global burden of disease. Recent estimates suggest that as many as 6.7 million global deaths per year can be attributed to air pollution, with most of these deaths related to cardiovascular disease.^1^ There is substantial evidence for a robust association between short‐term air pollution exposure and ischemic heart disease,^2^, ^3^ and there is some evidence of an association with cardiac arrhythmias, but it is less well understood.^2^, ^4^ Given the high incidence of cardiac arrhythmias, establishing an association with air pollution would provide more evidence to support that a reduction in air pollution levels would have substantial benefits for public health.

Health effects of low‐level air pollution exposure has gained considerable interest in Europe and North America, and key studies have demonstrated increased risk for cardiovascular disease^5^ and all‐cause death^6^, ^7^, ^8^ after long‐term exposure. Out‐of‐hospital cardiac arrest (OHCA) is one of the leading causes of death in the world,^9^ with approximately 300 000 fatal cases in Europe, of which 6000 cases occur in Sweden^10^ every year,^11^, ^12^ and despite considerable efforts, the 30‐day survival remains low.^13^ Several recent studies of short‐term exposure have reported associations between particulate matter (PM) <2.5 μm (PM_2.5_) and OHCA, with a 2017 meta‐analysis estimating an increased risk of 4% per 10 μg/m^3^.^14^ However, the studies contributing to the meta‐analysis were all conducted in areas with PM_2.5_ levels above the new World Health Organization (WHO) Air Quality Guidelines of 15 μg/m^3^ for daily means. Furthermore, previous studies investigating short‐term associations between air pollution and OHCA have relied on monitoring data, often located in highly polluted areas and in cities, limiting the ability to investigate the impacts on health of low levels of PM_2.5_ or in rural areas. At present, Sweden has one of Europe's lowest levels of PM_2.5_, with mean levels rarely exceeding the new daily WHO guideline of 15 μg/m^3^.^15^ With this nationwide study in Sweden we aim to investigate if PM_2.5_ as well as PM <10 μm (PM_10_), O_3_ and NO_2_ are associated with a higher risk of OHCA, even at low concentrations.

## Methods

### Data Availability Statement

The data that support the findings of this study are available from the corresponding author upon reasonable request and approval by the Swedish Ethical Review Authority.

### Study Population

We used data from the SRCR (Swedish Registry for Cardiopulmonary Resuscitation) described in detail elsewhere.^16^ In short, the SRCR is a national quality registry of OHCAs in which cardiopulmonary resuscitation has been started by either a health professional or bystander, and it complies with the Utstein style of reporting for all variables and outcomes.^17^ Since 2009, all emergency medical services in Sweden report data to the registry. Detailed information on time and location of the cardiac arrest as well as clinical characteristics such as first ECG rhythm, age, and sex are available. The emergency medical services‐personnel categorizes the cardiac arrest into medical and nonmedical (ie, traumatic, intoxication, asphyxiation, and drowning) cardiac arrests. This study included all medical cardiac arrests in the registry from 2009 to 2019. Cases with missing information on location or date of cardiac arrest were excluded. The address of pickup of the patient with cardiac arrest was geocoded and used as the location for exposure assessment.

### Exposure Assessment

We used a national machine learning model for exposure assessment for estimating daily levels of PM_2.5_, PM_10_, O_3_, NO_2_, and air temperature in a 1×1‐km grid all over Sweden. The model combines satellite remote‐sensing data (aerosol optical depth, land surface temperature, greenness, and light at night), information on land‐use variables and air pollution sources, and atmospheric models to predict air pollution and temperature levels using machine learning techniques. The model has been described in detail elsewhere^18^ and has been shown to have a high cross‐validation accuracy, for example, PM_2.5_ (cross‐validation R^2^=0.69), O_3_ (cross‐validation R^2^=0.77), and air temperature (cross‐validation R^2^ = 0.97).

The geocoded address of pick up for each case of OHCA was linked to the corresponding 1×1‐km grid cell and daily air pollution levels were estimated for the case and control days for that location.

### Statistical Analysis

We used a case‐crossover design with a time‐stratified referent selection strategy^19^, ^20^, ^21^ to investigate the association between air pollution levels and OHCA. Specifically, for each individual case of OHCA, exposure just before the event is compared with 3 to 4 control periods by matching on the same day of the week, month, and year as the case period. Potential confounders that are relatively stable over time are controlled for by the design through within‐participant comparisons.

The association between the different air pollutants and OHCA was quantified using conditional logistic regression and expressed as an odds ratio per 10 μg/m^3^ increase of air pollutant levels. The results are presented as percentage change in relative risk, calculated as (odds ratio – 1) × 100. The associations were adjusted for air temperature modeled with 2 natural splines to capture a potential nonlinear association between air temperature and OHCA: one for lag 0 to 1 (3 degrees of freedom, only for days above the median), aimed at capturing the immediate effects of high temperatures, and another for lag 1 to 6 (2 degrees of freedom, only for days below the median) aimed at capturing the cumulative effects of low temperatures.^22^ We investigated different individual day exposure windows for all 4 pollutants ranging from same‐day exposure (lag 0) to 6 days before (lag 6) in both single‐pollutant and multipollutant models with PM, O_3_, and NO_2_ in the same model in an approach toward obtaining independent effect estimates that account for potential confounding effects by other pollutants. The spring and summer periods were used as the main analysis for O_3_ because of the clear seasonality of O_3_ as it is a secondary pollutant created by the photochemistry conversion of NO_2_ to O_3_ in the presence of sunlight.

In secondary analyses, we stratified by season to evaluate potential seasonal differences in the association. We subsequently stratified the cases by first ECG rhythm to explore a potential difference in association between cases with first ECG rhythm of ventricular tachycardia (VT)/ventricular fibrillation (VF) or asystole/pulseless electrical activity (PEA). We analyzed the association between PM_2.5_ and OHCA for each stratum and investigated a potential interaction by creating multiplicative terms. We explored potential differences between cases occurring in urban or rural municipalities as defined by the Swedish Agency for Growth Policy Analysis.^23^ Further, we also explored potential effect modification by sex and age (only for the lag of PM_2.5_ with the most pronounced associations). Effect modification was investigated by adding interaction terms between the effect modifiers and the exposure, and by extracting stratum‐specific risk estimates and CIs. An effect modification was considered statistically significant if the *P* value of the interaction term was <0.05.

To test the robustness of the results, we conducted a series of sensitivity analyses. First, we modeled the different lags in a constrained distributed lag model (all lag terms modeled together and their coefficients constrained to follow a polynomial shape). This was done to try to account for the lag‐specific effects on the odds of OHCA accounting for potential confounding by other lagged effects. Then, to explore potential exposure misclassification, we restricted the analyses to cases occurring at home. We further conducted an analysis adjusting the association between air pollutants and OHCA for air temperature as a natural spline with 3 degrees of freedom with a common lag 0 to 6 for the full temperature range.

For all statistical testing, a 2‐sided *P* value of <0.05 was considered statistically significant. All statistical analyses were conducted with R version 4.1.1 (R Foundation for Statistical Computing, Vienna, Austria). This study was approved by the Swedish Ethical Review Authority and Stockholm regional ethical board (Dnr 2021‐02463 and 2009/1919‐31/1) and complied with the Declaration of Helsinki. Informed consent was not required.

## Results

**Figure 1:**
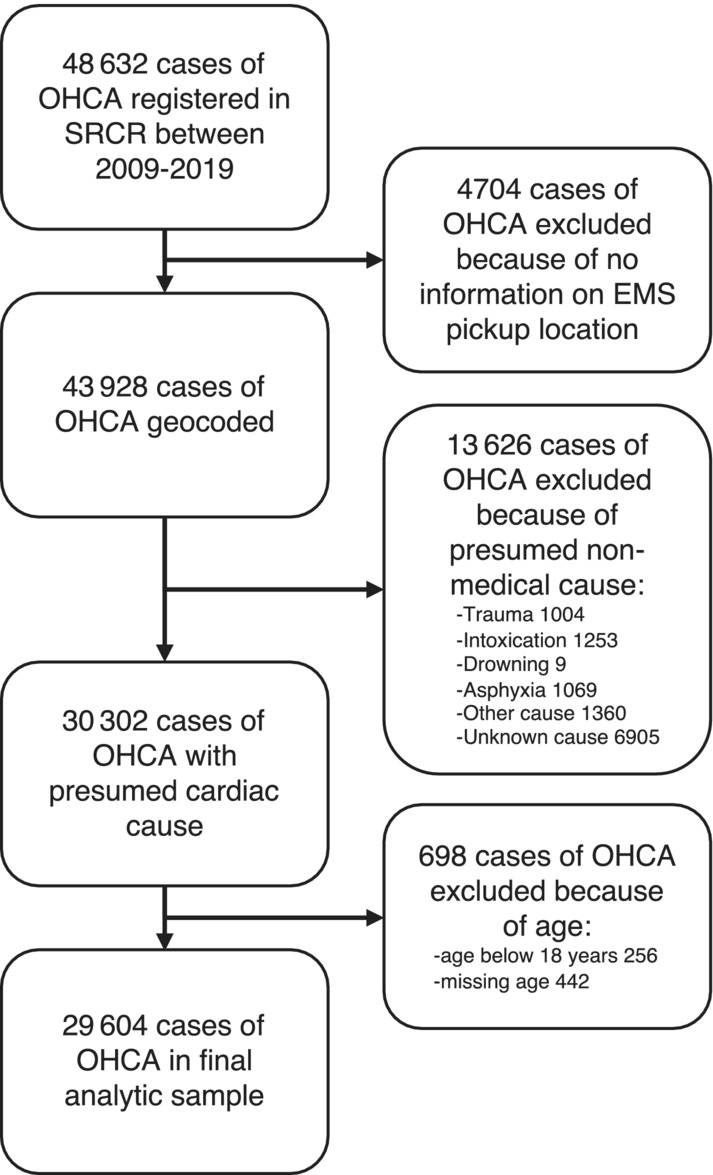
Flowchart of participant inclusion. EMS indicates emergency medical services; OHCA, out‐of‐hospital cardiac arrest; and SRCR, Swedish Registry for Cardiopulmonary Resuscitation.

**Figure 2:**
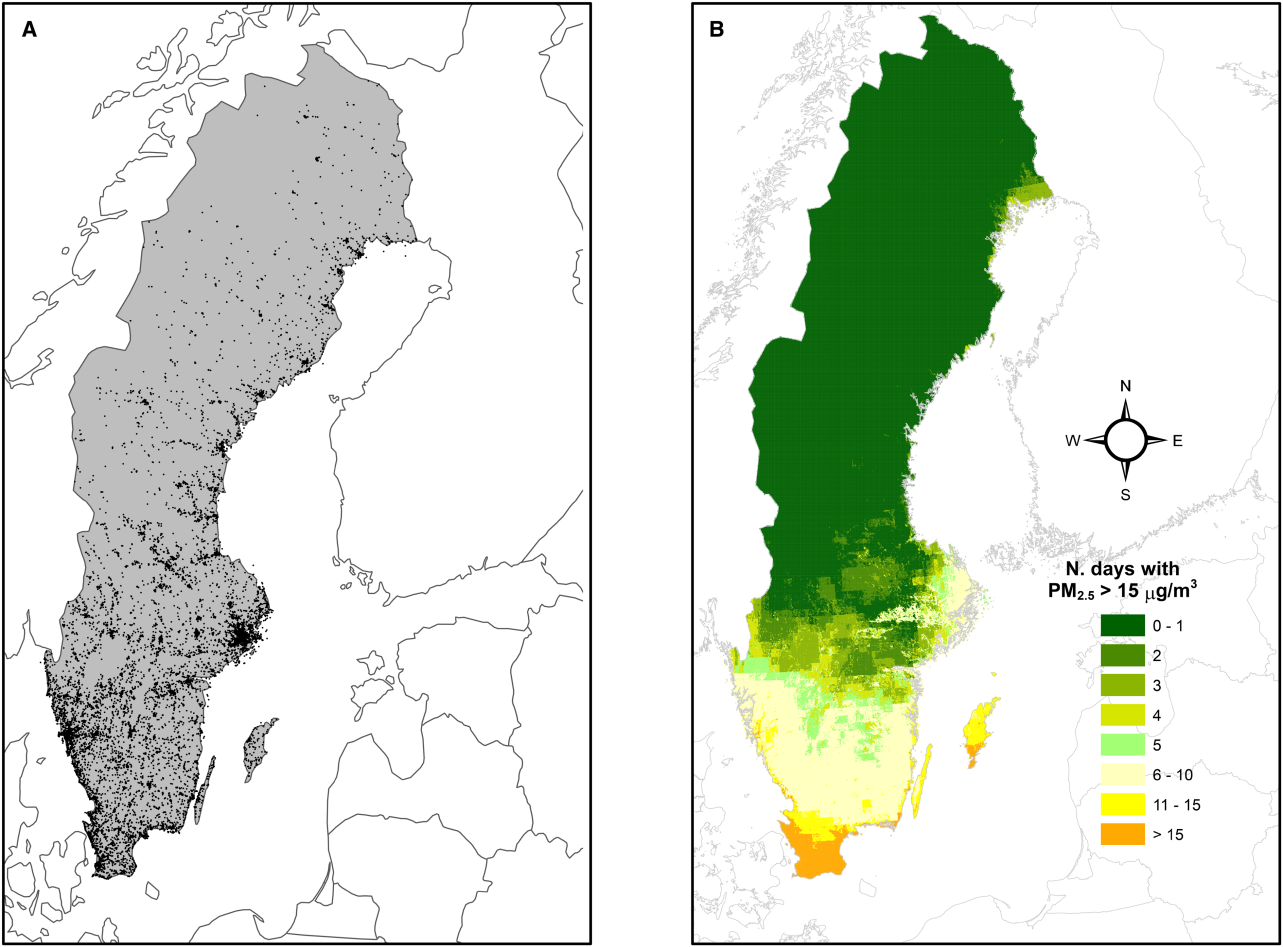
Distribution of OHCA during the study period 2009 to 2019 with 1 dot per case (A); and days exceeding the WHO guidelines of 15 μg/m^3^ PM2.5 during 2015 in the study area (B). OHCA indicates out‐of‐hospital cardiac arrest; and PM_2.5_, particulate matter <2.5 μm.

Between 2009 and 2019, 48 632 cases of cardiac arrest were registered in the SRCR. The final analytic sample consisted of 29 604 cases of OHCA after exclusion of cases with missing information on pickup, nonmedical cases, and age <18 years. A flowchart of included and excluded cases in the study population is presented in Figure 1. For the analytic sample, the median age was 74 years, and almost two‐thirds were men. A total of 21 322 (72%) of the cases occurred at home. There seemed to be a seasonal variation in the incidence of OHCAs, with the highest frequency during winter months (December through February) and a slight increase of cases over the years (Figure S1). Demographic and clinical characteristics are presented in Table 1. The SRCR has a national coverage, and this is reflected in Figure 2A, with cases from all over Sweden, albeit with more cases in the more densely populated areas in southern Sweden. As many as 64% of the cases occurred in municipalities classified as urban.

**Table 1:** Demographic and Clinical Characteristics

Characteristics	N=29 604
Age	
Median (IQR)	74 (65–83)
Sex, n (%)	
Men	20 019 (68)
Women	9583 (32)
Missing	2
Municipality type, n (%)	
Rural	18 859 (64)
Urban	10 745 (36)
Location of OHCA, n (%)	
Home	21 322 (72)
Public place	4886 (17)
Other location	3387 (11)
Missing	9
First ECG, n (%)	
VT/VF	7729 (27)
Asystole/PEA	21 437 (73)
Missing	438
Season, n (%)	
Winter	8135 (27)
Spring	7468 (25)
Summer	6786 (23)
Autumn	7215 (24)

OHCA indicates out‐of‐hospital cardiac arrest; PEA, pulseless electrical activity; and VT/VF, ventricular tachycardia/ventricular fibrillation. Winter: Dec, Jan, Feb. Spring: Mar, Apr, May. Summer: Jun, Jul, Aug. Autumn: Sep, Oct, Nov.

The exposure model had complete data for the study period. PM_10_ and O_3_ showed some variation over the different seasons, whereas we did not observe any clear difference over the year for PM_2.5_ and NO_2_. The daily median PM_2.5_ level was 7.2 μg/m^3^ with an interquartile range (difference between third and first quartiles) of 3.7. Overall and seasonal levels of air pollutants and air temperature are presented in Table 2, and distribution of the different pollutants are supplied in Figure S2. We observed a geographic gradient of PM_2.5_ from higher levels in the south to lower in the north. The number of days exceeding the WHO daily guidelines of 15 μg/m^3^ for 1 year are presented in Figure 2B. PM_2.5_ and PM_10_ were highly correlated (correlation coefficient, 0.80), while the remaining pollutants did not show strong correlations. NO_2_ and O_3_ were modestly negatively correlated (−0.26). Correlations are presented in Table S1, and seasonal correlations are presented in Table S2.

**Table 2:** Daily Air Pollution and Air Temperature Levels

Pollutant	Overall*	Winter*	Spring*	Summer*	Autumn*
PM_2.5_, μg/m^3^	7.2 (5.6–9.3)	7.3 (5.8 to 9.2)	8.5 (6.5–10.6)	7.1 (5.6–9.0)	6.3 (4.9–8.1)
PM_10_, μg/m^3^	13.7 (10.7–17.9)	12.7 (10.0 to 16.2)	18.3 (13.6–24.2)	13.4 (11.0–16.2)	12.2 (9.4–15.6)
O_3_, μg/m^3^	54.2 (44.3–63.8)	48.3 (40.3 to 55.8)	67.4 (60.9–73.0)	57.8 (51.9–63.9)	43.1 (36.5–50.3)
NO_2_, μg/m^3^	11.4 (6.7–16.9)	14.2 (8.7 to 21.1)	11.5 (6.8–16.6)	9.1 (4.6–12.6)	11.4 (6.9–16.5)
Temp, °C	6.3 (1.1–13.4)	−0.4 (−4.0 to 2.4)	5.7 (2.1–9.7)	16.2 (14.5–17.9)	7.5 (4.1–11.4)

NO_2_ indicates nitrogen dioxide; O_3_, ozone; PM_10_, particulate matter <10 μm; PM_2.5_, particulate matter <2.5 μm; and Temp, air temperature.

* Median (interquartile range).

**Figure 3:**
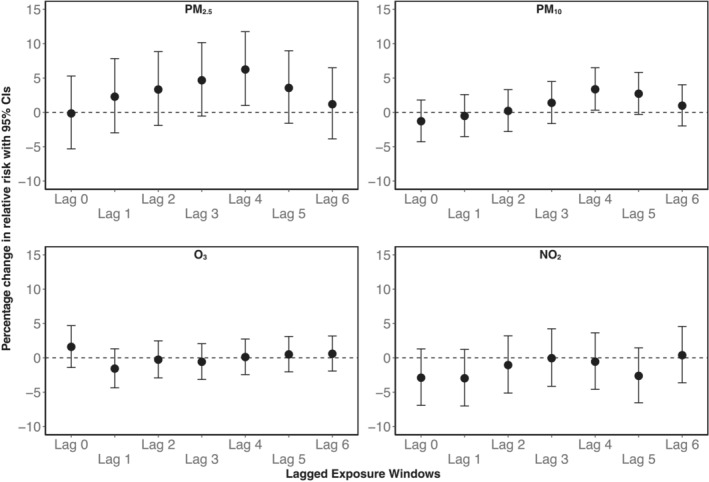
Multipollutant model of the association between daily lagged air pollutant exposure windows and OHCA as percentage change in relative risk per 10 μg/m^3^ increase with 95% CIs adjusted for daily air temperature as natural splines (lag 0–1 for days with temperature above the median and lag 1–6 for days below the temperature median). Lag estimates are provided for same day (lag 0) and each preceding daily average 6 days before event. PM_2.5_, PM_10_, NO_2_ analyses included all days across all seasons, while O_3_ were restricted to spring and summer (Mar–Sep) only. PM_2.5_ and PM_10_ risk estimates are adjusted for O_3_ and NO_2_. O_3_ and NO_2_ risk estimates are adjusted for PM_2.5_. OHCA indicates out‐of‐hospital cardiac arrest; PM_10_, particulate matter <10 μm; and PM_2.5_, particulate matter <2.5 μm.

In the multipollutant model, we observed a delayed association between PM_2.5_ and OHCA, with higher risk associated with increasing levels in our main analysis. The strongest point estimates were observed for lag 3 and lag 4. The percentage change in relative risk for lag 4 was 6.2% (95% CI, 1.0–11.8) per 10 μg/m^3^ increase. PM_10_ and PM_2.5_ showed similar patterns in lagged associations, but point estimates were less pronounced for PM_10_ (Figure 3). We did not observe any clear associations between O_3_ nor NO_2_ and OHCA for the lagged exposure windows. The single‐pollutant models showed similar results for PM_2.5_ and PM_10_. For the gaseous pollutants, we observed positive associations for O_3_ on lag but with a large degree of uncertainty. For NO_2_, we observed a counterintuitive negative association for lag 0 with no clear pattern for longer lags. Single‐pollutant models are presented in Figure S3.

We explored potential seasonal differences for all 4 pollutants. As described in the Methods section, our main model included days from the full year for all pollutants except O_3_, where the main model was restricted to cases occurring during the season with the highest levels, that is, spring and summer. We did not observe any clear differences in the associations during the different seasons for PM_2.5_, PM_10_, nor NO_2_. For O_3_, we observed more pronounced associations for the stratified analysis of winter compared with the spring and summer, with statistically significant associations for lag 0 (Table S3). Similarly, the whole‐year analysis for ozone showed statistically significant associations for lag 0 for the single‐lag model (Table S4).

**Figure 4:**
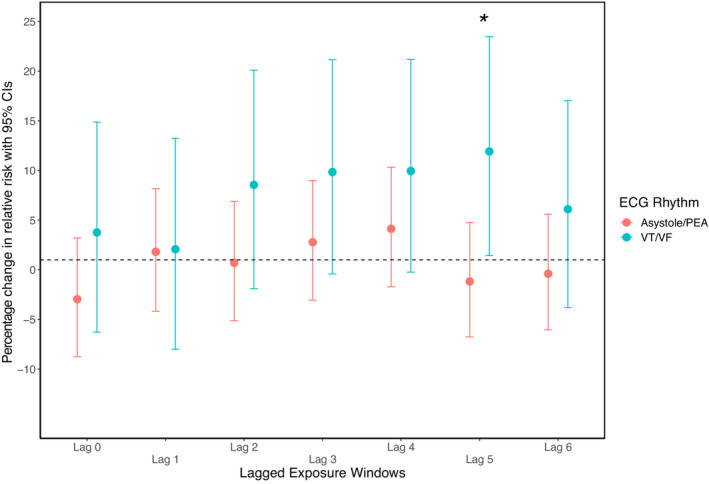
Associations between PM_2.5_ exposure windows and OHCA stratified by first recorded ECG rhythm. Daily lagged estimates are provided as percentage change in relative risk per 10 μg/m^3^ with 95% CIs for same day (lag 0) and each preceding daily average 6 days before event. All estimates are adjusted for daily air temperature as natural splines (lag 0–1 for days with temperature above the median and lag 1–6 for days below the temperature median). OHCA indicates out‐of‐hospital cardiac arrest; PEA, pulseless electrical activity; PM_2.5_, particulate matter <2.5 μm; and VT/VF, ventricular tachycardia/ventricular fibrillation. *P value for interaction lag 5=0.036.

In the stratified analysis, we observed stronger risk estimates for cases with first ECG rhythm of VT/VF compared with cases with asystole/PEA (Figure 4). The risk estimates were positive for all lags for VT/VF with the strongest estimates in lag 2 to lag 5, with a statistically significant interaction for lag 5 (*P* value for interaction=0.036). For asystole/PEA cases, the pattern was less clear, with no significant associations, and not as pronounced risk estimates for the intermediate lags (lag 3, lag 4) compared with the VT/VF group despite including considerably more cases. We did not observe effect modification by type of area with similar risk estimates for lag 4 of PM_2.5_ for rural and urban cases with a *P* value for interaction of 0.89 (risk estimate: rural, 5.6% [95% CI, −3.2 to 14.9]; and urban, 6.2% [95% CI, −2.5 to 15.7]). We further explored potential susceptible groups and interactions by sex and age. For lag 4 PM_2.5_ exposure, we observed stronger risk estimates for women (10.6% [95% CI, 1.7–20.3]) compared with men (3.6% [95% CI, −4.7 to 12.7]), although the interaction was not statistically significant. We did not observe an interaction by age above and below the median (*P* value for interaction=0.641).

In sensitivity analyses, all pollutants showed similar association patterns for both single lags and distributed lags (Figure S4). The sensitivity analyses restricting the data set to cases with reported location of cardiac arrest that occurred at home showed similar associations as the main model for all 4 pollutants with a delayed association and strongest risk estimate for lag 4 for PM2.5 (Figure S5). Adjusting the associations to a common lag (lag 0–6) for the full temperature range showed similar results as the main approach. Results presented in Table S5.

## Discussion

In this Swedish national case‐crossover study among 29 604 cases of OHCAs between 2009 and 2019 we observed a higher risk associated with short‐term increases in spatially modeled PM_2.5_ levels. The association was most pronounced for lag 3 and lag 4, and associations were more apparent for OHCAs with the first ECG rhythm of VT or VF compared with asystole or PEA. Associations between PM_10_ and OHCA were similar but with smaller relative risk estimates.

Results for gaseous pollutants were less clear. We observed a positive but nonstatistically significant association for same‐day exposure for O_3_ and OHCA during summer months, broadly consistent with our previous study, which found statistically significant associations for same‐day O_3_ in Stockholm, Gothenburg, and Malmö,^24^ and a meta‐analysis from 2017 reporting associations between O_3_ exposure and OHCA.^14^ Although we observed a counterintuitive negative association between NO_2_ levels and OHCAs, this was attenuated and no longer statistically significant in the 2‐pollutant model with O_3_, perhaps partially due to their negative correlation as an expected consequence of O_3_ being a secondary pollutant created from the reaction of nitrogen oxide and O_2_ in the presence of sunlight.

The observed positive association between short‐term PM_2.5_ and OHCA is in line with several previous studies.^25^, ^26^, ^27^, ^28^, ^29^, ^30^, ^31^, ^32^, ^33^, ^34^, ^35^, ^36^ We report an increased risk of 6% per 10 μg/m^3^ increase for PM_2.5_, stronger than 4%, the result of Zhao and colleagues’ 2017 meta‐analysis, but within the 95% CI, ranging from 1% to 7%.^14^ A few recent studies from Korea and Japan have reported risk estimates ranging from 1.6% to 4% per 10 μg/m^3^ increase of PM_2.5._ ^25^, ^26^, ^27^, ^28^ The different studies have investigated different exposure windows, and direct comparisons therefore might not be straightforward. However, a pattern similar to the one reported in this study with delayed association was observed in a few studies,^28^, ^30^, ^32^ while several studies^25^, ^26^, ^27^, ^29^, ^31^, ^33^, ^34^, ^35^, ^36^ observed associations on the same day. Differences in observed results for exposure windows as well as for the strength of the association might be partly explained by different study populations and air pollution characteristics, sources, levels, and exposure assessment methods.

Compared with most of the previous studies, our study was conducted in the region of generally low levels of air pollution with, for example, an annual mean of 6.6 μg/m^3^ in 2019 and a daily median of 7.2 μg/m^3^. Interestingly, we observed similar risk estimates for PM_2.5_ exposure and OHCAs occurring in urban and rural areas. Consequently, our results do not support that PM would be less harmful in a rural setting despite potentially different particle compositions, socioeconomic status, lifestyle, and access to health care as well as relatively lower levels.^37^ Rather, our findings suggest associations to be relevant even in nonurban areas. Our findings are especially important considering the new stricter WHO air quality guidelines from 2021 recommending daily means <15 μg/m^3^ and the currently ongoing revision of the current EU air quality directive with more permissive standards still pending.

Given the heterogeneity in the potential pathophysiological causes of cardiac arrest, the findings of a potentially stronger association for cases with VT/VF compared with asystole/PEA cases is of mechanistical interest. For cardiac OHCAs, the most common pathophysiological pathway in is an initial VT degenerating first to VF and later to asystole.^38^ Noncardiac OHCAs are more heterogeneous and tend to have a larger proportion of asystole/PEA as the primary rhythm.^39^ A stronger association with VT/VF cases might indicate a direct arrhythmogenic effect of PM.

It is recognized that ≈80% of cardiac OHCAs are related to ischemic heart disease either from an acute thrombotic event or related to an anatomic substrate (scarring from previous myocardial infraction) without current ischemia. To a lesser extent, the OHCA could result from a cardiomyopathy or ion‐channel defect, valvular disease, or congenital heart diseases.^38^, ^40^ Ischemia is considered the most common triggering factor of OHCA, but OHCAs can also be triggered by systemic metabolic and hemodynamic alterations, neurophysiological factors, and exogenous effects. The triggering mechanism can in turn interact with the underlying pathophysiological increased risk of, for example, ischemia, anatomic substrate, or ion‐channel deficiencies.^38^, ^40^ The pathophysiological pathway that might connect air pollution exposure to arrhythmias is not completely established. There is, however, some evidence of air pollutants, and foremost PM, having an adverse health effect by local inflammation in the lungs that cascades into a systemic inflammation, translocation in the bloodstream with local inflammation and oxidative stress, and effects on the autonomous nervous system.^2^, ^41^ Both inflammation and autonomic dysfunction could promote ventricular arrhythmias and are plausible pathways for the PM effect. There is evidence from animal studies of increased risks of premature ventricular contractions^42^, ^43^ and ventricular arrhythmias^42^ with PM exposure. Further, studies in humans have reported PM exposure and increased risk of premature ventricular contractions,^44^ prolonged QT interval,^45^ and repolarization disturbances^46^, ^47^ (ECG changes that might be a precursor to ventricular arrhythmias). Several epidemiological studies have shown increased risk of VT/VF episodes in patients with implantable cardioverter‐defibrillators within hours of air pollution exposure.^48^, ^49^ Our findings in this study suggested a lagged or delayed association more in line with an inflammatory cascade initiated by PM inhalation, but a clear pathophysiological pathway is still unclear and is probably multifactorial.

The results from this study must be interpreted in the context of several limitations. The exposure was assigned on the basis of emergency medical services pickup location. This introduces potential exposure misclassification with participants potentially assigned an exposure for an area they only briefly visited. The magnitude of the exposure misclassification is expected to be larger for analyses of lags longer than the same day. However, we did not see a substantial difference in analyses conducted that were restricted to cases that occurred only at home, where we expect the exposure misclassification to be less important. Further, there is always a question of potential exposure misclassification when ambient rather than personal exposure to air pollution is used, but this misclassification should be nondifferential on average and tends to bias results toward the null hypothesis of no association. Another limitation is the lack of information on personal risk factors for cardiovascular disease such as previous medical conditions, exercise, or smoking. However, the case‐crossover design, with comparisons within individuals, means that risk factors that are relatively stable over time are not expected to confound our analyses but could be important in evaluating potential susceptible groups and investigating who is at risk of OHCA with increased air pollution levels. Future studies could investigate potential risk factors and vulnerable groups by using information from registries with patient medical status and history as well as personal behaviors such as exercise and smoking habits. Further, it would be of interest to investigate the potential synergistic effects of air pollutants and air temperature.

On the other hand, this study has several important strengths. The large sample size with cases of OHCA from all of Sweden combined with a sophisticated ensemble exposure model enabled us to investigate the association between air pollution and OHCA in a low‐pollution area that has not been studied before, as well as in both rural and urban areas and not limited to where monitor data have been available. Further, the model provides estimates with high temporal and spatial resolution, reducing exposure misclassification by assigning exposure on 1×1‐km grids. The use of a prospective national registry gives a robust outcome assessment, and the high coverage reduces the risk of selection bias.

In conclusion, in this national case‐crossover study including almost 30 000 OHCA from across Sweden and spanning over a decade, we found a higher risk of OHCAs associated with short‐term exposure to PM_2.5_ and PM_10_. Our findings add to the evidence of an adverse effect of PM on OHCA even at low levels below current regulatory guidelines.

## Sources of Funding

This work was supported by the Swedish Research Council for Health, Working Life and Welfare (grant number 2015‐00917) to M.D. and P.L.S.L.; and Karolinska Institute's Strategic Research Area in Epidemiology to P.L.S.L.

## Disclosures

Dr Wellenius serves as a consultant to the Health Effects Institute (Boston, MA) and Google, LLC (Mountain View, CA). The remaining authors have no disclosures to report.

## Supporting information

Tables S1–S5
Figures S1–S5
