# Supplementary material for: Short‐Term Ambient Air Pollution Exposure and Risk of Out‐of‐Hospital Cardiac Arrest in Sweden: A Nationwide Case‐Crossover Study
Source: J Am Heart Assoc. 2023 Oct 11;12(21):e030456. doi: 10.1161/JAHA.123.030456 (PMC10727387; doi:10.1161/JAHA.123.030456)
Supplement: Supplementary file 1 — Tables S1–S5 Figures S1–S5 [file JAH3-12-e030456-s001.pdf]

Supplemental Material

Table S1. Air pollution and air temperature correlations*

	$PM_{2.5}$	PM_{10}	O_3	NO_2	Temperature
$PM_{2.5}$					
PM_{10}	0.80				
O_3	0.07	0.20			
NO_2	0.28	0.39	-0.34		
Temperature	0.04	0.06	0.27	-0.26	

**Pearson Correlation Coefficients, $PM_{2.5}$: Particulate matter <2.5μm, PM_{10}: Particulate matter < 10μm, O_3: Ozone, NO_2: Nitrogen Dioxide*

Table S2. Air pollution correlations* by season

**Winter**	$PM_{2.5}$	PM_{10}	O_3	NO_2	Temperature
$PM_{2.5}$					
PM_{10}	0.85				
O_3	-0.12	-0.07			
NO_2	0.26	0.33	-0.49		
Temperature	0.00	0.08	0.12	-0.18	

**Spring**	$PM_{2.5}$	PM_{10}	O_3	NO_2	Temperature
$PM_{2.5}$					
PM_{10}	0.73				
O_3	-0.14	-0.13			
NO_2	0.32	0.56	-0.44		
Temperature	0.10	0.05	0.20	-0.09	

**Summer**	$PM_{2.5}$	PM_{10}	O_3	NO_2	Temperature
$PM_{2.5}$					
PM_{10}	0.86				
O_3	0.33	0.36			
NO_2	0.30	0.49	-0.10		
Temperature	0.43	0.49	0.46	0.17	

**Autumn**	$PM_{2.5}$	PM_{10}	O_3	NO_2	Temperature
$PM_{2.5}$					
PM_{10}	0.90				
O_3	-0.13	-0.14			
NO_2	0.27	0.43	-0.38		
Temperature	0.27	0.26	0.29	-0.15	

**Pearson Correlation Coefficients, OHCA: Out-of-Hospital Cardiac Arrest, OR: Odds Ratio, $PM_{2.5}$: Particulate matter < 2.5μm, PM_{10}: Particulate matter < 10μm, O_3: Ozone, NO_2: Nitrogen Dioxide, Winter: Dec, Jan, Feb, Spring: Mar, Apr, May, Summer: Jun, Jul, Aug, Autumn: Sep, Oct, Nov*

Table S3. Association between single daily lags of air pollution levels and OHCA per 10 µg/m^3 stratified by season. All estimates are adjusted for daily air temperature as natural splines (lag 0-1 for days with temperature above the median and lag 1-6 for days below the temperature median).

	**Winter**	**Spring**	**Summer**	**Autumn**
**Exposure**	OR (95% CI)	OR (95% CI)	OR (95% CI)	OR (95% CI)
$PM_{2.5}$ lag 0	0.97 (0.89, 1.06)	1.01 (0.91, 1.12)	1.09 (0.92, 1.29)	0.93 (0.84, 1.03)
$PM_{2.5}$ lag 1	1.02 (0.94, 1.11)	1.04 (0.94, 1.15)	1.01 (0.86, 1.19)	0.98 (0.88, 1.08)
$PM_{2.5}$ lag 2	1.04 (0.96, 1.14)	1.03 (0.94, 1.13)	0.98 (0.85, 1.14)	1.02 (0.92, 1.13)
$PM_{2.5}$ lag 3	1.06 (0.97, 1.16)	1.09 (1.00, 1.20)	0.95 (0.82, 1.10)	1.01 (0.92, 1.12)
$PM_{2.5}$ lag 4	1.05 (0.96, 1.15)	1.07 (0.99, 1.17)	1.02 (0.88, 1.18)	1.06 (0.97, 1.17)
$PM_{2.5}$ lag 5	1.04 (0.95, 1.14)	1.01 (0.93, 1.11)	0.95 (0.82, 1.09)	1.06 (0.96, 1.17)
$PM_{2.5}$ lag 6	1.06 (0.96, 1.16)	0.96 (0.88, 1.05)	0.95 (0.82, 1.10)	1.05 (0.96, 1.16)
PM_{10} Lag 0	0.96 (0.91, 1.01)	1.01 (0.96, 1.05)	1.03 (0.92, 1.15)	0.95 (0.89, 1.01)
PM_{10} Lag 1	1.00 (0.95, 1.05)	0.99 (0.95, 1.04)	0.99 (0.89, 1.10)	0.97 (0.90, 1.03)
PM_{10} Lag 2	1.00 (0.94, 1.05)	1.00 (0.96, 1.05)	1.03 (0.93, 1.13)	1.00 (0.94, 1.06)
PM_{10} Lag 3	1.02 (0.97, 1.08)	1.02 (0.98, 1.07)	1.00 (0.91, 1.10)	1.00 (0.94, 1.06)
PM_{10} Lag 4	1.04 (0.98, 1.10)	1.03 (0.99, 1.08)	1.02 (0.93, 1.11)	1.03 (0.97, 1.10)
PM_{10} Lag 5	1.03 (0.97, 1.09)	1.01 (0.97, 1.06)	0.99 (0.90, 1.08)	1.04 (0.98, 1.10)
PM_{10} Lag 6	1.03 (0.97, 1.09)	1.00 (0.96, 1.05)	1.02 (0.93, 1.12)	1.00 (0.94, 1.06)
NO_2 Lag 0	0.93 (0.89, 0.98)	0.91 (0.84, 0.98)	1.13 (0.98, 1.31)	1.01 (0.94, 1.09)
NO_2 Lag 1	1.00 (0.95, 1.05)	0.96 (0.89, 1.04)	1.00 (0.86, 1.16)	0.97 (0.9, 1.05)
NO_2 Lag 2	0.99 (0.94, 1.04)	0.98 (0.91, 1.06)	1.00 (0.86, 1.16)	0.98 (0.91, 1.06)
NO_2 Lag 3	0.99 (0.95, 1.04)	1.03 (0.96, 1.11)	1.06 (0.92, 1.23)	0.98 (0.91, 1.06)
NO_2 Lag 4	1.01 (0.96, 1.06)	0.98 (0.91, 1.05)	1.14 (0.98, 1.32)	0.96 (0.89, 1.04)
NO_2 Lag 5	0.99 (0.95, 1.04)	1.00 (0.93, 1.08)	0.96 (0.83, 1.11)	0.94 (0.87, 1.02)
NO_2 Lag 6	1.01 (0.97, 1.06)	1.00 (0.93, 1.08)	1.08 (0.93, 1.25)	1.00 (0.92, 1.08)
O_3 Lag 0	1.04 (1.01, 1.06)	1.03 (1.00, 1.07)	1.01 (0.96, 1.05)	1.00 (0.97, 1.04)
O_3 Lag 1	1.00 (0.98, 1.03)	1.00 (0.97, 1.04)	0.98 (0.94, 1.02)	1.00 (0.97, 1.03)
O_3 Lag 2	1.02 (0.99, 1.05)	1.01 (0.97, 1.04)	0.99 (0.96, 1.03)	1.01 (0.98, 1.04)
O_3 Lag 3	1.01 (0.98, 1.03)	1.00 (0.96, 1.03)	0.99 (0.95, 1.02)	1.01 (0.98, 1.04)
O_3 Lag 4	0.99 (0.97, 1.02)	1.02 (0.99, 1.06)	0.98 (0.95, 1.02)	1.02 (0.99, 1.05)
O_3 Lag 5	1.00 (0.98, 1.03)	1.02 (0.98, 1.05)	0.99 (0.96, 1.03)	1.01 (0.97, 1.04)
O_3 Lag 6	0.99 (0.96, 1.01)	1.01 (0.98, 1.04)	0.99 (0.96, 1.03)	1.00 (0.97, 1.04)

OHCA: Out-of-Hospital Cardiac Arrest, OR: Odds Ratio, $PM_{2.5}$: Particulate matter < 2.5µm, PM_{10}: Particulate matter < 10µm, O_3: Ozone, NO_2: Nitrogen Dioxide, Winter: Dec, Jan, Feb, Spring: Mar, Apr, May, Summer: Jun, Jul, Aug, Autumn: Sep, Oct, Nov

Table S4. Association between daily lagged (Lag 0, same day to Lag 6, six days prior to event) ozone exposure windows and OHCA as Odds Ratios per 10 μg/m^3 increase with 95% confidence intervals. All estimates are adjusted for daily air temperature as natural splines (lag 0-1 for days with temperature above the median and lag 1-6 for days below the temperature median).

	**Single lag model**	**Distributed lag model**
**Exposure**	OR (95% CI)	OR (95% CI)
O_3 Lag 0	1.023 (1.007, 1.039)	1.011 (0.998, 1.024)
O_3 Lag 1	0.999 (0.984, 1.015)	1.006 (1.000, 1.013)
O_3 Lag 2	1.011 (0.995, 1.027)	1.003 (0.997, 1.009)
O_3 Lag 3	1.003 (0.988, 1.019)	1.001 (0.994, 1.008)
O_3 Lag 4	1.005 (0.990, 1.020)	0.999 (0.993, 1.006)
O_3 Lag 5	1.004 (0.989, 1.019)	0.999 (0.994, 1.005)
O_3 Lag 6	0.996 (0.982, 1.011)	1.000 (0.989, 1.013)

OHCA: Out-of-Hospital Cardiac Arrest, OR: Odds Ratio, $PM_{2.5}$: Particulate matter < 2.5μm, PM_{10}: Particulate matter < 10μm, O_3: Ozone, NO_2: Nitrogen Dioxide

Table S5. Single pollutant model of the association between daily lagged air pollutant exposure windows and OHCA as percent change in relative risk per 10 μg/m^3 increase with 95% confidence intervals adjusted for daily air temperature as a natural spline (lag 0-6 for all cases). Lag estimates are provided for same day (Lag 0) and each preceding daily average six days prior to event. $PM_{2.5}$, PM_{10}, NO_2 analyses included all days across all seasons while O_3 were restricted to spring and summer (Mars-September) only.

**Exposure**	OR (95% CI)
$PM_{2.5}$ Lag 0	0.978 (0.930, 1.029)
$PM_{2.5}$ Lag 1	1.011 (0.961, 1.063)
$PM_{2.5}$ Lag 2	1.026 (0.975, 1.080)
$PM_{2.5}$ Lag 3	1.051 (0.999, 1.106)
$PM_{2.5}$ Lag 4	1.070 (1.018, 1.125)
$PM_{2.5}$ Lag 5	1.038 (0.986, 1.092)
$PM_{2.5}$ Lag 6	1.023 (0.973, 1.076)
PM_{10} Lag 0	0.977 (0.950, 1.006)
PM_{10} Lag 1	0.988 (0.959, 1.017)
PM_{10} Lag 2	1.001 (0.972, 1.030)
PM_{10} Lag 3	1.018 (0.988, 1.048)
PM_{10} Lag 4	1.038 (1.009, 1.069)
PM_{10} Lag 5	1.027 (0.998, 1.057)
PM_{10} Lag 6	1.016 (0.988, 1.045)
O_3 Lag 0	1.023 (0.995, 1.051)
O_3 Lag 1	0.993 (0.967, 1.020)
O_3 Lag 2	1.001 (0.977, 1.026)
O_3 Lag 3	0.993 (0.969, 1.017)
O_3 Lag 4	1.004 (0.980, 1.028)
O_3 Lag 5	1.005 (0.981, 1.029)
O_3 Lag 6	1.001 (0.977, 1.024)
NO_2 Lag 0	0.953 (0.921, 0.987)
NO_2 Lag 1	0.982 (0.948, 1.017)
NO_2 Lag 2	0.983 (0.949, 1.018)
NO_2 Lag 3	1.001 (0.967, 1.037)
NO_2 Lag 4	0.998 (0.964, 1.033)
NO_2 Lag 5	0.981 (0.948, 1.015)
NO_2 Lag 6	1.009 (0.975, 1.044)

OHCA: Out-of-Hospital Cardiac Arrest, OR: Odds Ratio, $PM_{2.5}$: Particulate matter < 2.5μm, PM_{10}: Particulate matter < 10μm, O_3: Ozone, NO_2: Nitrogen Dioxide

Figure S1. Daily count of out-of-hospital cardiac arrest cases (black line) and 30-day rolling averages divided by season for the study period.

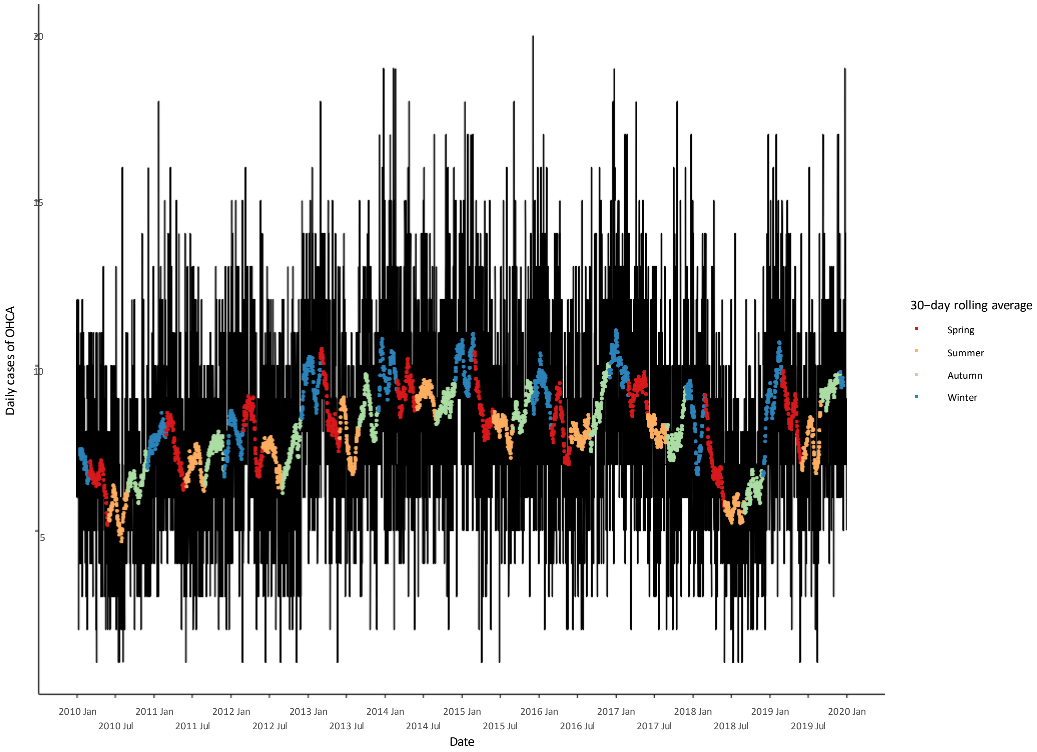

OHCA: Out-of-Hospital Cardiac Arrest

Figure S2. Four-panel figure with distributions of air pollutants ($PM_{2.5}$, PM_{10}, O_3, and NO_2) stratified by season.

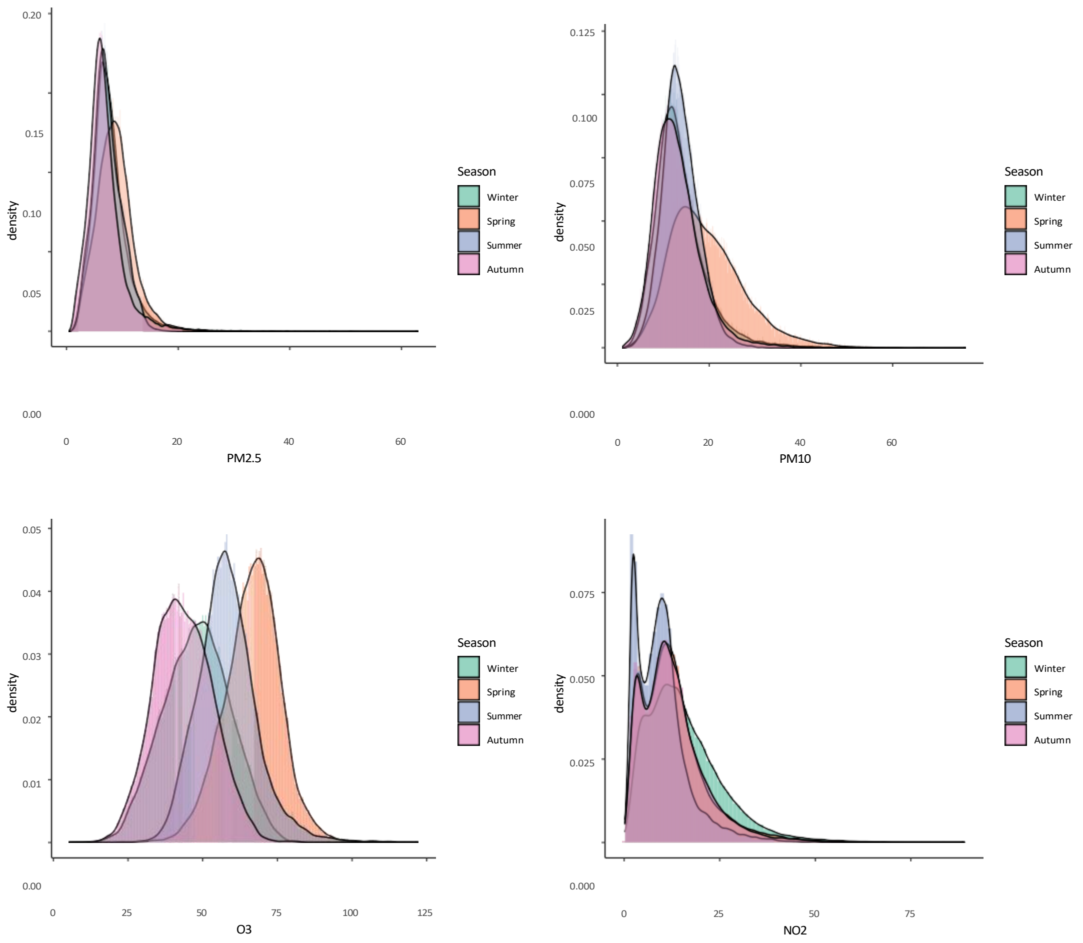

PM2.5: Particulate matter <2.5µm, PM10: Particulate matter <10µm, O3: Ozone, NO2: Nitrogen Dioxide Winter: Dec, Jan, Feb, Spring: Mar, Apr, May, Summer: Jun, Jul, Aug, Autumn: Sep, Oct, Nov

Figure S3. Single pollutant model of the association between daily lagged air pollutant exposure windows and OHCA as percent change in relative risk per 10 μg/m³ increase with 95% confidence intervals adjusted for daily air temperature as natural splines (lag 0-1 for days with temperature above the median and lag 1-6 for days below the temperature median). Lag estimates are provided for same day (Lag 0) and each preceding daily average six days prior to event. $PM_{2.5}$, PM_{10}, NO_2 analyses included all days across all seasons while O_3 were restricted to spring and summer (Mars-September) only.

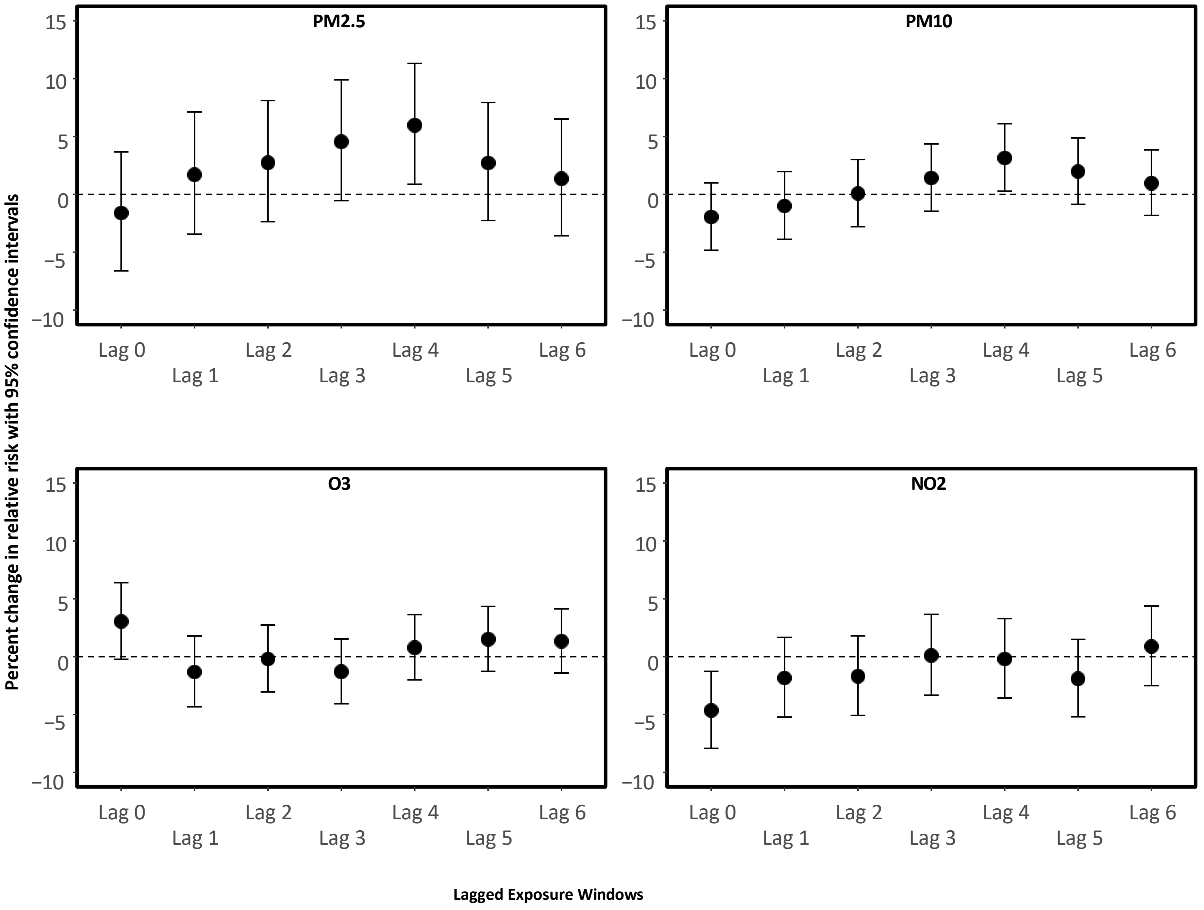

OHCA: Out-of-Hospital Cardiac Arrest, $PM_{2.5}$: Particulate matter < 2.5μm, PM_{10}: Particulate matter < 10μm, O_3: Ozone, NO_2: Nitrogen Dioxide

Figure S4. Association between daily lagged (Lag 0, same day to Lag 6, six days prior to event) air pollutant exposure windows and OHCA as Odds ratios per 10 μg/m^3 increase with 95% confidence intervals. Black dots are derived from a single lag model and blue from a constricted distributed lag model. $PM_{2.5}$, PM_{10}, NO_2 is whole year while O_3 is restricted to Warm season (Mar-Sep) only. All estimates are adjusted for daily air temperature as natural splines (lag 0-1 for days with temperature above the median and lag 1-6 for days below the temperature median).

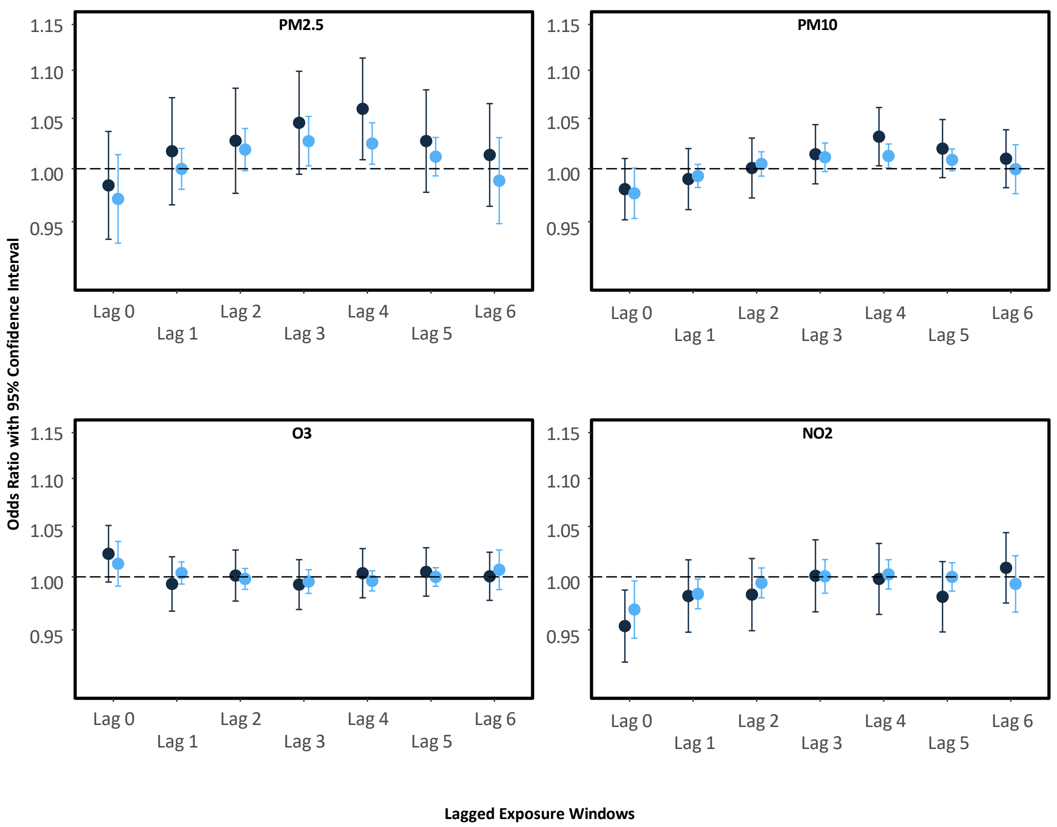

OHCA: Out-of-Hospital Cardiac Arrest, $PM_{2.5}$: Particulate matter < 2.5μm, PM_{10}: Particulate matter < 10μm, O_3: Ozone, NO_2: Nitrogen Dioxide

Figure S5. Stratified analysis including only cases that occurred at home. Association between daily lagged (Lag 0, same day to Lag 6, six days prior to event) air pollutant exposure windows and OHCA as Odds ratios per 10 μg/m^3 increase with 95% confidence intervals. Black dots are derived from a single lag model and blue from a constricted distributed lag model. $PM_{2.5}$, PM_{10}, NO_2 is whole year while O_3 is restricted to Spring and Summer (Mar-Sep) only. All estimates are adjusted for daily air temperature as natural splines (lag 0-1 for days with temperature above the median and lag 1-6 for days below the temperature median).

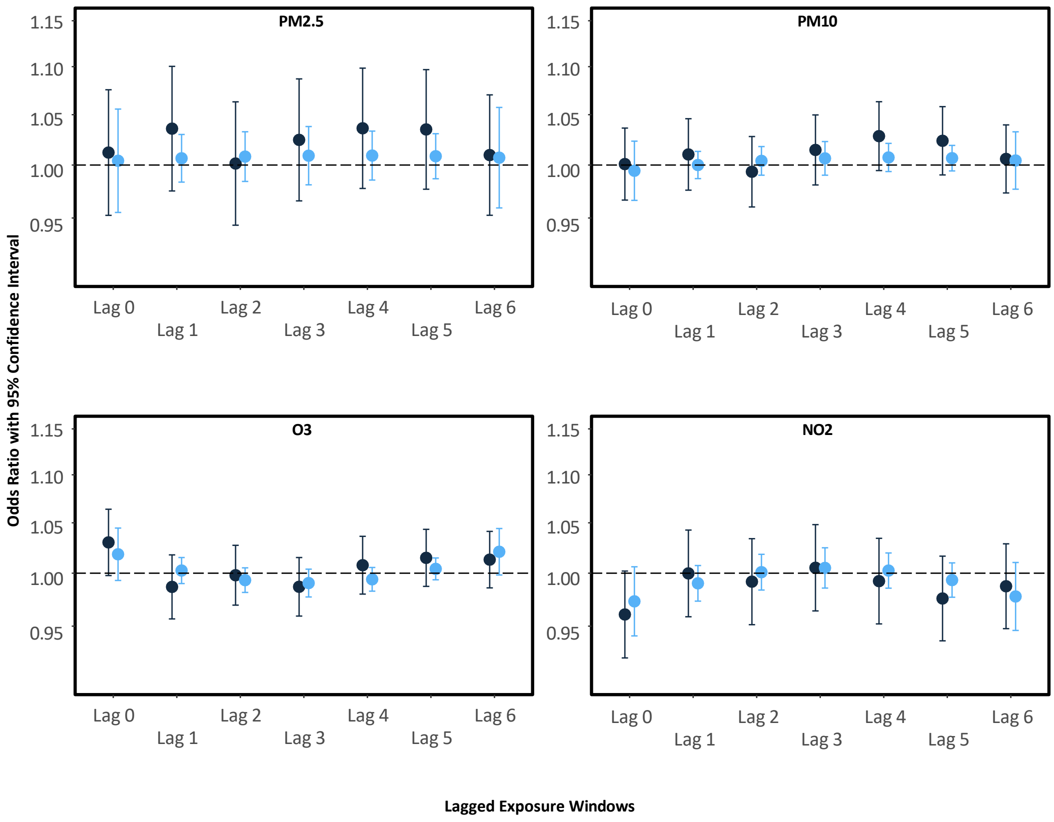

OHCA: Out-of-Hospital Cardiac Arrest, $PM_{2.5}$: Particulate matter < 2.5µm, PM_{10}: Particulate matter < 10µm, O_3: Ozone, NO_2: Nitrogen Dioxide
